# Molecular Dynamics Simulations Reveal that Water Diffusion between Graphene Oxide Layers is Slow

**DOI:** 10.1038/srep29484

**Published:** 2016-07-08

**Authors:** Ram Devanathan, Dylan Chase-Woods, Yongsoon Shin, David W. Gotthold

**Affiliations:** 1 Pacific Northwest National Laboratory, Richland, Washington 99352, USA

## Abstract

Membranes made of stacked layers of graphene oxide (GO) hold the tantalizing promise of revolutionizing desalination and water filtration if selective transport of molecules can be controlled. We present the findings of an integrated study that combines experiment and molecular dynamics simulation of water intercalated between GO layers. We simulated a range of hydration levels from 1 wt.% to 23.3 wt.% water. The interlayer spacing increased upon hydration from 0.8 nm to 1.1 nm. We also synthesized GO membranes that showed an increase in layer spacing from about 0.7 nm to 0.8 nm and an increase in mass of about 15% on hydration. Water diffusion through GO layers is an order of magnitude slower than that in bulk water, because of strong hydrogen bonded interactions. Most of the water molecules are bound to OH groups even at the highest hydration level. We observed large water clusters that could span graphitic regions, oxidized regions and holes that have been experimentally observed in GO. Slow interlayer diffusion can be consistent with experimentally observed water transport in GO if holes lead to a shorter path length than previously assumed and sorption serves as a key rate-limiting step.

Graphene oxide (GO) has potential applications in opto-electronics, energy storage, solar cells, biomedical technologies, and membranes for selective molecular separation^1^,^2^,^3^,^4^,^5^,^6^. In particular, there is growing interest in GO membranes that allow rapid transport of water for desalination, water purification, dehumidification and filtration^7^,^8^,^9^,^10^. Such interest is driven by the reality that more than a billion people worldwide suffer from an acute shortage of clean water and human activity continues to pollute fresh water resources^11^. While technologies exist for waste water treatment and seawater desalination, they are inefficient, energy intensive, expensive, and environmentally unsustainable^12^. GO-based membranes hold the promise of cost-effective water filtration, because they can be rationally designed and synthesized using an inexpensive and scalable process to selectively permit certain molecules or ions to pass through.

One of the key challenges to realizing the potential of GO membranes for selective separations is the lack of mechanistic understanding of molecular interactions and transport. Even basic details, such as the structure of GO, are the subject of considerable debate and several models have been proposed to explain the structure of GO^5^,^13^,^14^,^15^,^16^,^17^,^18^. The most commonly invoked description of the chemical structure of GO is the Lerf-Klinowski model^19^ and the structure is known to vary with synthesis conditions and degree of oxidation^5^. GO is considered to be amorphous and non-stoichiometric^5^ with hydroxyls and epoxides as the dominant functional groups along with carbonyls at the edges of the platelets^16^. High resolution transmission electron microscopy (HRTEM) studies by Erickson *et al*.^16^ have revealed that the GO sheet has holes, with a typical size of 5 nm^2^ or less, a continuous oxidized network, and isolated graphitic regions. The GO sheet is known to be rippled with surface roughness of at least 0.6 nm^13^. It is a daunting task to characterize H_2_O transport in GO while accounting for this heterogeneity and disorder.

Recently, the Geim group has reported^7^ extraordinarily rapid permeation of H_2_O molecules through submicrometer thick GO membranes, which show He permeation rates that are slower than H_2_O permeation rates by a factor of 10^10^. The authors attributed the anomalously rapid transport of H_2_O molecules to capillary pressure of the order of 100 MPa while a monolayer of H_2_O molecules flows through a network of graphene capillaries with spacing between 0.6 and 1 nm. A subsequent report^8^ by the same group showed that ions with hydrated radii less than 0.45 nm permeated rapidly through GO membranes by the same mechanism. A neutron scattering study by Buchsteiner *et al*.^20^ has shown that the layer spacing of graphite oxide multilayers varies from about 0.7 to 1.1 nm with increasing humidity, the interlayer spacing and H_2_O uptake are affected by the synthesis method, and the spaces between layers could be filled with varying levels of water. This study also found that the interlayer H_2_O molecules were bound by hydrogen bonds to the oxygen of epoxide and hydroxyl groups at almost all hydration levels and bulk-like water occurred only at the highest hydration levels.

A different interpretation of H_2_O transport in GO membranes is provided by Talyzin *et al*.^21^. These authors observed the GO interlayer spacing to increase from about 0.8 to 1.2 nm following immersion in liquid water. In light of the aforementioned HRTEM studies by Erickson *et al*.^16^ that graphene-like regions in GO are isolated, which is also evidenced by the poor electrical conductivity of GO, Talyzin *et al*.^21^ have argued that the extraordinarily rapid water transport inferred by Nair *et al*.^7^ is an interpretation based on an excessively long and tortuous migration path that does not account for the presence of defects and holes. Since permeation of H_2_O through GO will involve oxidized and unoxidized regions, Talyzin *et al*.^21^ recommended that the flow of H_2_O molecules be modeled between two hydrophilic surfaces representing the structure of GO instead of two hydrophobic surfaces.

Given the experimental challenges of unambiguously characterizing water transport in the disordered environment of GO membranes, molecular modeling has a key role to play in the interpretation of experimental observations. Several molecular dynamics (MD) simulation studies have focused on water interactions with graphene^7^,^8^,^22^,^23^,^24^. There is a pressing need to use computer simulation to study GO-water interactions. Boukhvalov and Katsnelson^25^ used density functional theory (DFT) to optimize the structure of GO and suggested the chemical formulas C_8_(OH)_2_, C_8_(OH)_2_O and C_8_(OH)_4_O for coverage of 25%, 50 and 75%, respectively. The spacing in a bilayer at 25% coverage was found to be 0.7 nm. A DFT study by Yan *et al*.^26^ found that significant energy gains were attained if the OH groups formed 1,2-hydroxyl pairs on opposite sides of the GO sheet and certain combinations of epoxide and hydroxyl groups were also energetically favored. A subsequent DFT study^27^ by the same group yielded optimized structures with values of bond lengths, bond angles, and displacements of oxygen atoms from the plane of graphene.

MD simulations^15^,^28^ using reactive force fields that allow bond breaking and bond formation have shed light on hydrogen bonds between H_2_O molecules and functional groups in GO and the evolution of GO structure during thermal annealing. Due to the computational intensity of reactive force fields, these studies have been restricted to sample sizes of about 4 nm × 4 nm and simulation times of the order of tens of picoseconds. Wei *et al*.^29^ performed MD simulations of H_2_O transport in GO with carbon atoms frozen in a planar conformation for specific interlayer distances and showed that the H_2_O flow is affected by electrostatic interactions in GO. Raghav *et al*.^30^ calculated the potential of mean force for small graphene or GO sheets with 84 carbon atoms constrained as parallel layers at different separations and immersed in H_2_O or He, and showed that GO layers respond dynamically to their environment. Ban *et al*.^31^ performed MD simulations of rigid H_2_O permeation in a rigid GO bilayer system for fixed layer separation along with experimental x-ray photoelectron spectroscopy, calorimetry and sorption measurements. The H_2_O diffusion coefficient in GO for layer separations greater than 1 nm calculated in this study was surprisingly higher than that in bulk H_2_O.

In an effort to understand the molecular mechanisms that control H_2_O transport through GO membranes, we have performed MD simulations of model GO multilayers intercalated with H_2_O for different hydration levels. These simulations have provided information about bound H_2_O, radial distribution functions, and H_2_O diffusion coefficients as a function of hydration level. The simulations were accompanied by experimental solution filtration and casting of GO membranes to provide information about interlayer spacing and mass increase with hydration. The present study shows that the diffusion of H_2_O molecules between GO layers is too slow to account for the experimentally inferred rapid transport of water through GO membranes.

## Results and Discussion

Table S1 in the Supplementary Information presents the density of the GO simulation cell at different hydration levels and it was in the range from 0.96 to 1.29 g/cm^3^. The layer spacing varied from 0.8 nm in GO without water to 1.1 nm in GO with intercalated water regardless of water content from 1 to 23 wt%. This change in layer spacing is consistent with the experimental results of Talyzin *et al*.^21^ Our experimental results in Figure S1(a) show that the inter-layer spacing decreased steadily from about 0.78 nm to 0.69 nm upon vacuum drying for 9 days. On subsequent rehydration over 10 days, this spacing increased steadily from 0.69 nm to about 0.79 nm. The corresponding variation in the mass of the membrane from 10.2 mg to 8.8 mg on drying and back to 10.2 mg on rehydration can be seen in Figure S1(b). Figure S1(c) reveals that the membrane thickness decreased from 9 μm to 7 μm after drying for 9 days and increased back to 9 μm after rehydration for 10 days. The variation of the thickness gives the appearance of being non-monotonic because of the limitation of the measurement technique in that the measurements were in steps of 1 μm. Our simulation results for the layer spacing are also in good agreement with the neutron scattering results of Lerf *et al*.^32^, who observed the layer spacing in graphite oxide to vary from 0.8 to 1.15 nm as the relative humidity (RH) increased from 45 to 100%. Experimental hydration levels are often expressed in terms of RH^20^,^32^, while the wt.% value from our simulation is the ratio of the mass of water to the mass of water and membrane expressed as a percent. Buchsteiner *et al*.^20^ have pointed out that RH of 75% corresponds to the uptake of a monolayer of water in the interlayer spacing. Our experimental results show that the change in mass from a fully hydrated membrane to a membrane vacuum dried for 9 days is about 15%, which can be attributed to the water removed. Even allowing for some bound water retained by the membrane during vacuum drying, the range of water content (1 to 23 wt.%) simulated in the present work covers the experimentally relevant hydration levels.

**Figure 1:**
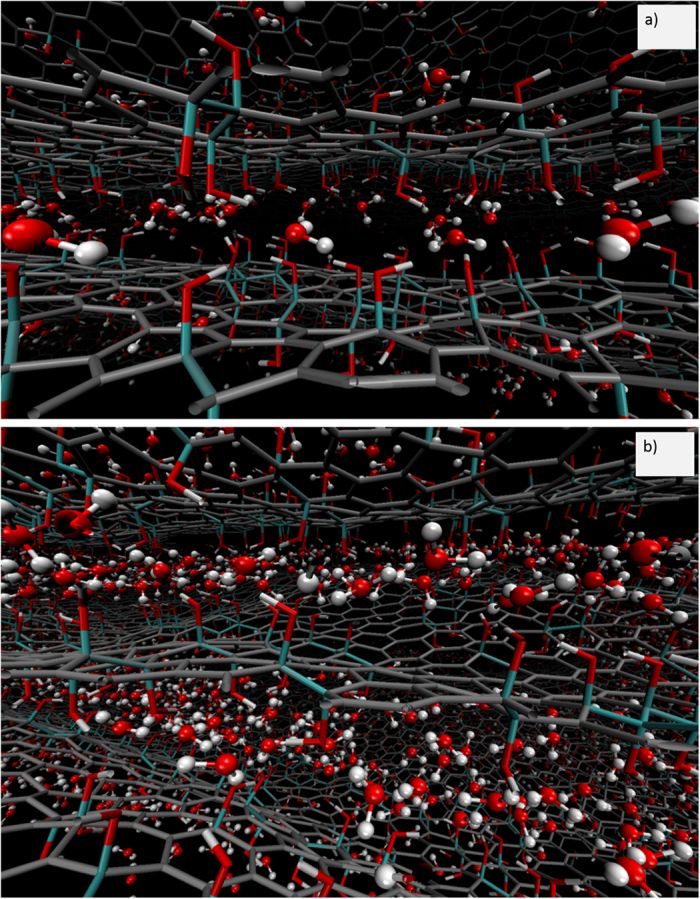
Graphene oxide configurations for (**a**) 8.3 wt.% and (**b**) 23.3 wt.% H_2_O. O, H, C bonded to OH and other C are shown in red, white, teal and grey, respectively.

Figure 1 shows a snapshot of atom positions from simulations with 8.3 wt.% H_2_O and 23.3 wt.% H_2_O. The hydroxyl groups are clustered and there are graphene-like patches between these clustered OH units. Our simple model of GO, C_4_(OH), has hydroxyl groups only and does not include epoxide groups or holes. The GO layers are corrugated because the atoms were not frozen and were allowed to relax. At the lower hydration level, the H_2_O molecules are isolated and found close to OH groups, while at the highest hydration level, clusters of H_2_O molecules are evident. Isolated H_2_O molecules are seen even more clearly at 3.9 wt.% H_2_O in Figure S2 of the Supplementary Information. These findings are entirely consistent with the results from reactive force field MD studies of Medhekar *et al*.^28^ at a comparable C/O ratio—C_10_O_1_(OH)_1_—for water content from 1 to 26 wt.%.

**Figure 2:**
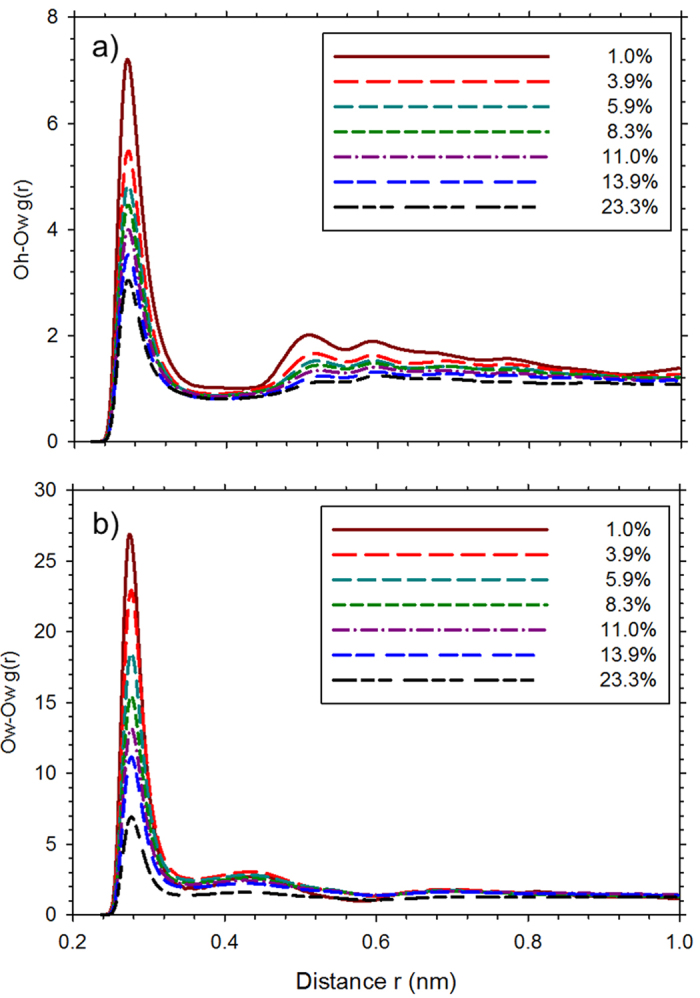
Pair correlation functions between (**a**) oxygen atoms of OH group and H_2_O and (**b**) oxygen atoms of H_2_O for the water content shown in the legend.

More evidence about water interactions in GO can be seen in the pair correlation functions between OH group oxygen (O_h_) and water oxygen (O_w_) and that between O_w_ and O_w_ presented in Fig. 2 for different hydration levels. The O_h_-O_w_ distribution has a sharp first peak at 0.27 nm that indicates strong binding of the H_2_O molecules to the OH group. With increasing hydration level, the height of this peak decreases and the longer range structure (second and subsequent peaks) starts to disappear, which shows that H_2_O molecules move away from the OH groups. A similar trend is seen in the O_w_-O_w_ pair correlation function that shows a first peak at 0.275 nm, minimum at 0.35 nm and a second peak at 0.43 nm. With increasing hydration level, the O_w_-O_w_ distribution approaches that of liquid water, which shows a peak value of 3.1 at 0.288 nm^33^. The inference is that H_2_O molecules behave more like those in bulk water as the hydration level increases. The location of the O_h_-O_w_ and O_w_-O_w_ first peaks is at a shorter separation compared to the simulation results of Medhekar *et al*.^28^ that show an optimum 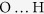 bond distance of 0.255 nm for two H_2_O molecules in GO connected by a hydrogen bond. To shed more light on hydrogen bonding in our GO-H_2_O system, we have calculated the pair correlation functions for the hydrogen of OH (H_o_) and O_w_ and for O_h_ and the hydrogen of H_2_O (H_w_). These are plotted in Figures S3 and S4, respectively, of the Supplementary Information. In both these cases, the first peak is at 0.17 nm and the first minimum is at 0.24 nm, which is completely consistent with the oxygen-oxygen distance of about 0.27 nm in our work.

**Figure 3:**
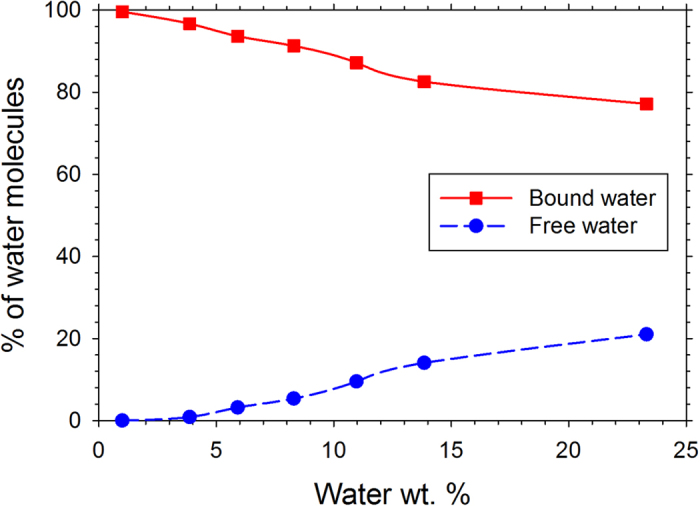
Percent of bound (square) and free (circle) H_2_O as a function of water wt.% in graphene oxide.

**Figure 4:**
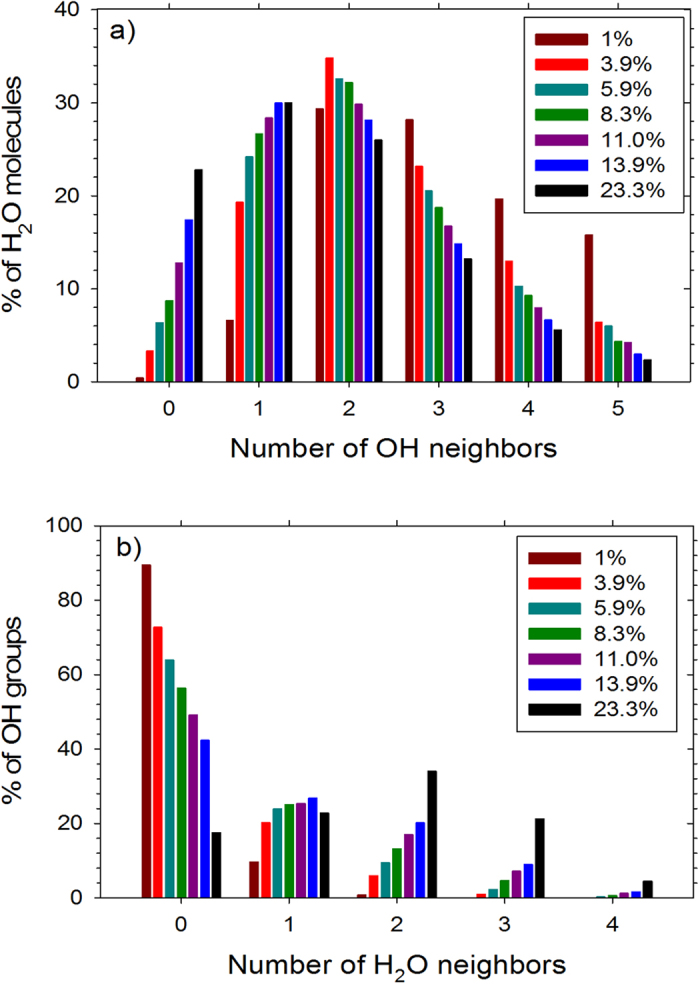
(**a**) Distribution of OH groups near H_2_O molecules and (**b**) distribution of H_2_O molecules near OH groups based on a cutoff distance of 0.35 nm between O_h_ and O_w_.

Figure 3 is a plot of the percent of H_2_O molecules that are bound to OH groups or free (bulk-like) at various hydration levels. The details of these calculations are given in the Methods section. About 1–3% of H_2_O molecules do not fit into either of these categories and can be considered nearly bound. Almost all the H_2_O molecules are ‘bound’ to OH groups at the lowest hydration level of 1 wt.% H_2_O. Our term ‘bound water’ includes both bound and confined water in the neutron scattering study of Buchsteiner *et al*.^20^ while our ‘free water’ is similar to the ‘bulk water’ determined by these authors^20^. The percent of free H_2_O molecules increases with increasing hydration level and reaches about 21% at the highest hydration level studied. We have shown the distribution of OH groups within a distance of 0.35 nm from H_2_O molecules in Fig. 4(a) and the distribution of H_2_O molecules within a distance of 0.35 nm from OH groups in Fig. 4(b). At the highest hydration level (23.3 wt.% H_2_O), the average H_2_O molecule is most likely to have one OH neighbor, while at lower hydration levels it is most likely to have two OH neighbors. The model of water dynamics in graphite oxide from neutron scattering^20^ includes H_2_O molecules hydrogen bonded to multiple functional groups. Except at the highest hydration level a large proportion of OH groups do not have H_2_O molecules within a distance of 0.35 nm, which is due to the fact that the number of OH groups (8230) is greater than the number of H_2_O molecules in those cases (see Table S1 of the Supplementary Information for details). These distributions could change if the C/O ratio is changed or if epoxide groups are introduced. Nonetheless, these findings shed light on the strong hydrogen bonding between the OH groups and H_2_O molecules. Our results are in good agreement with the finding of the neutron scattering study of water in graphite oxide^20^ that water is tightly bound except at the highest humidity level studied.

**Figure 5:**
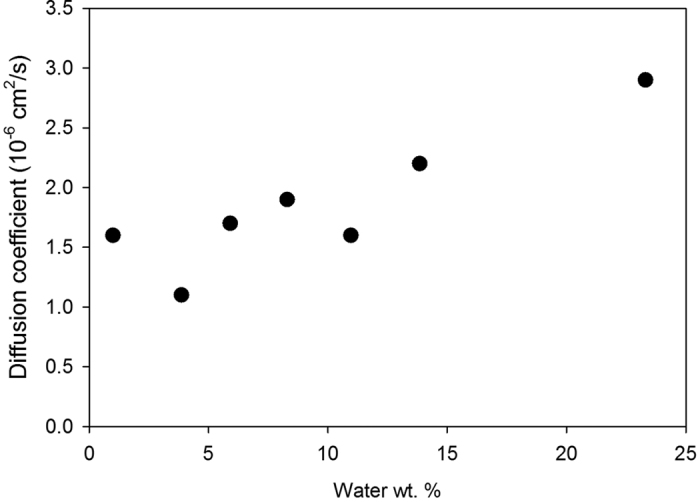
The diffusion coefficient of water as a function of hydration level in graphene oxide.

The effect of hydration level on the water diffusion coefficient (*D*_*w*_) in GO is presented in Fig. 5. *D*_*w*_ shows a general increasing trend with increasing hydration level although there is some scatter in the data. These values are an order of magnitude less than the diffusion coefficient in bulk water, which is to be expected given the strong hydrogen bonded interaction with OH groups at all hydration levels. Diffusion at the lowest hydration level, where almost all H_2_O molecules are bound, could still occur by migration between adjacent OH groups as suggested by Buchsteiner *et al*.^20^ based on neutron scattering.

As the water content increases, the percent of water molecules that are free increases substantially from about 3.2% at a hydration level of 5.9 wt.% water to 21% at 23.3 wt.% water. This increase results in faster diffusion, because a larger proportion of water molecules can move unhindered by OH groups. The average water cluster size increases with hydration level as shown in Figure S5 of the Supplementary information. If isolated water molecules (cluster size *n* = 1 molecule) are included, the average cluster size increases from 1.2 molecules for a hydration level of 1 wt.% water to 33.7 molecules for 23.3 wt.% water. If isolated molecules are excluded from the definition of a cluster, these numbers are 2.3 and 83.2 molecules, respectively. The largest H_2_O cluster size varied from 10 molecules at 1 wt.% water to 2948 molecules at 23.3 wt.% as shown in Table S2 of the Supplementary Information. Some of these clusters are large enough to extend from the oxidized region to the unoxidized regions or holes that have been experimentally observed.

The present simulations show strong hydrogen bonding between OH groups and H_2_O molecules resulting in slow water diffusion in GO, which has important implications for the recent interpretation by the Geim group^7^ that H_2_O molecules are transported at an extraordinarily rapid rate through GO membranes by graphene capillaries. Since it has been experimentally shown^16^ that graphitic regions in GO layers are isolated while the oxidized regions are continuous, fast transport through graphitic regions cannot entirely explain the observed rapid water permeation. Recently, Huang *et al*.^34^ examined the separation performance of GO for dimethyl carbonate (DMC)/water and methanol/water mixtures and the sorption of DMC, methanol and water on GO. They concluded that preferential transport of molecules through GO is influenced by both sorption and interlayer diffusion. In a subsequent study by the same group^35^, the water transport and selective separation of water from n-butanol was found to be enhanced when a hydrophilic polymer layer was deposited on the surface of GO laminates. Rapid water permeation was attributed to increased water sorption due to the hydrophilic surface and the availability of molecular channels within the GO laminates. A recent review of GO membranes^36^ has pointed that interlayer channels, defects or holes, and functional groups are important factors controlling molecular transport through GO.

Large water clusters, observed in the present work, could bridge graphitic and oxidized regions as well as holes. The graphitic regions could be isolated or extensive depending on the C/O ratio and the synthesis method. Rapid interlayer water transport is unlikely through the GO region, but could take place through a shorter path between graphitic regions via holes and spaces between flakes. The functional groups lining the holes and the hydrogen bonded network in the oxidized regions seen in the present study will hinder the transport of solvated ions and thus contribute to the observed selective transport. Further simulation of molecular sorption at the GO surface and transport of molecules and ions through GO containing holes and representative graphitic regions is needed to shed more light on selective transport in GO, especially for desalination.

## Conclusions

We have performed molecular dynamics simulations of water interactions in graphene oxide (GO) membrane at several hydration levels. We considered a model system with hydroxyl groups only and a C/O ratio of 4. Our results show that water diffusion in GO is an order of magnitude slower than in bulk water due to strong hydrogen bonded interactions between H_2_O molecules and the OH group. The optimum distance for the hydrogen bond (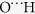) in our simulation is 0.17 nm and the oxygen-oxygen distance is typically 0.27 nm. Even at the highest hydration level of 23.3 wt.% H_2_O, only about 21% of the H_2_O molecules were free or bulk-like. We observed large water clusters comprising 10 to 30% of the water molecules present in the system. Such clusters can span across oxidized regions, graphitic regions, and defects or holes that have been seen in experiments thereby contributing to rapid water transport. Our results are in good agreement with results from neutron scattering studies of hydrated GO layers.

## Methods

We synthesized membranes from a range of different GO sources, both commercial and prepared at Pacific Northwest National Laboratory (PNNL), with the goal of obtaining various flake sizes, from 100 nm to 100 μm in nominal diameter. We used a simple solution filtration and casting on a polytetrafluoroethylene plate (12” × 12”) to isolate free-standing GO membranes. In addition to the variable flake sizes, we prepared membranes with thicknesses ranging from 5 μm to 50 μm and characterized them using multiple measurement techniques including X-ray diffraction pattern to detect the change in layer spacing that accompanies changes in mass and thickness of GO with changes in the hydration level.

We performed classical molecular dynamics simulations of GO membranes with H_2_O using the DLPOLY4 computer code^37^. We chose the composition C_8_(OH)_2_ suggested by Boukhvalov and Katsnelson^25^ and obtained the charges of the hydroxyl group from a restricted electrostatic potential fit following calculations at the B3LYP/6-31G* level of theory^38^,^39^ using the Gaussian 98 program^40^. The charges of O, H, and C bonded to the OH group in the present simulation are −0.53*e*, 0.35*e* and 0.18*e*, respectively, where *e* is the magnitude of the electron charge. All other carbon atoms are uncharged. We used the DREIDING^41^ force field to describe the GO layer, the F3C^42^ potential for H_2_O, and the non-bonded interaction parameters of Wu and Aluru^43^ for the C-H_2_O interactions. We used harmonic bond stretching and angle bending terms with bond lengths and bond angles constrained to correspond to the relaxed GO structure calculated by Yan and Chou^27^.

The simulation cell contained three layers of GO and was periodically repeated in all dimensions. We created GO layers with a C/O ratio of about 4 by randomly selecting two adjacent C atoms that were not already bonded to hydroxyl groups and attaching 1,2-hydroxyl pairs on opposite sides of the layer in a configuration optimized by Yan *et al*.^26^. The distribution of OH groups is different for each layer, and the three layers together had 33120 C atoms and 8230 OH groups. Starting with a layer spacing of 1.5 nm, we introduced a monolayer of H_2_O molecules between the GO layers with the number of H_2_O molecules ranging from 300 to 9075. We studied eight different hydration levels with water weight% varying from 0 to 23.9%. These values cover the range of H_2_O content seen in our experimental samples and presented in Figure S6 of the Supplementary Information. The GO-H_2_O system was annealed for 4 ns in the temperature range from 300 to 600 K as described previously^44^. We did not freeze any atom, fix the layer separation, fix the density of the system, or constrain the distribution of water molecules. After equilibration, the size of the simulation cell was about 17 nm × 17 nm × 3 nm.

We carried out the production run at constant volume and temperature of 300 K for 5 ns with a time step of 1 fs. We determined the H_2_O diffusion coefficient from the last 1 ns of data by performing a linear regression to the mean square displacement vs. time. We calculated the radial distribution functions for different pairs of atoms and obtained the number of bound H_2_O molecules by considering a molecule to be bound if the distance between water oxygen (O_w_) and OH group oxygen (O_h_) was less than 0.35 nm. A H_2_O molecule was considered to be free if that O_w_ was surrounded by four or more O_w_ within a distance of 0.4 nm. We also determined the extent of clustering of H_2_O molecules by considering two molecules to belong to the same cluster if the O_w_-O_w_ separation was less than 0.35 nm as discussed previously^44^,^45^,^46^.

## Additional Information

**How to cite this article**: Devanathan, R. *et al*. Molecular Dynamics Simulations Reveal that Water Diffusion between Graphene Oxide Layers is Slow. *Sci. Rep.* **6**, 29484; doi: 10.1038/srep29484 (2016).

## Supplementary Material

Supplementary Information
