# Supplementary material for: Molecular Dynamics Simulations Reveal that Water Diffusion between Graphene Oxide Layers is Slow
Source: Sci Rep. 2016 Jul 8;6:29484. doi: 10.1038/srep29484 (PMC4937448; doi:10.1038/srep29484)
Supplement: Supplementary Information [file srep29484-s1.doc]

**Molecular Dynamics Simulations Reveal that Water Diffusion between Graphene Oxide Layers is Slow**

Ram Devanathan*, Dylan Chase-Woods, Yongsoon Shin and David Gotthold

Pacific Northwest National Laboratory, Richland, Washington 99352

*Corresponding author: [ram.devanathan@pnnl.gov](mailto:ram.devanathan@pnnl.gov)

**Supplementary Information**

Table S1. Properties of the GO-water systems simulated.

| Wt. % H_2_O | # of H_2_O molecules | Layer spacing (nm) | Density (g/cc) |
| --- | --- | --- | --- |
| 0.0 | 0 | 0.80 | 1.29 |
| 1.0 | 300 | 1.09 | 0.96 |
| 3.9 | 1200 | 1.09 | 0.99 |
| 5.9 | 1875 | 1.09 | 1.01 |
| 8.3 | 2700 | 1.10 | 1.03 |
| 11.0 | 3675 | 1.10 | 1.05 |
| 13.9 | 4800 | 1.10 | 1.09 |
| 23.3 | 9075 | 1.09 | 1.25 |

Table S2. Largest water cluster size at each hydration level.

| Wt. % H_2_O | # of H_2_O molecules | Largest cluster size (molecules) | Fraction of total # of H_2_O molecules in largest cluster |
| --- | --- | --- | --- |
| 1.0 | 300 | 10 | 0.03 |
| 3.9 | 1200 | 60 | 0.05 |
| 5.9 | 1875 | 132 | 0.07 |
| 8.3 | 2700 | 328 | 0.12 |
| 11.0 | 3675 | 416 | 0.11 |
| 13.9 | 4800 | 1396 | 0.29 |
| 23.3 | 9075 | 2948 | 0.32 |

| 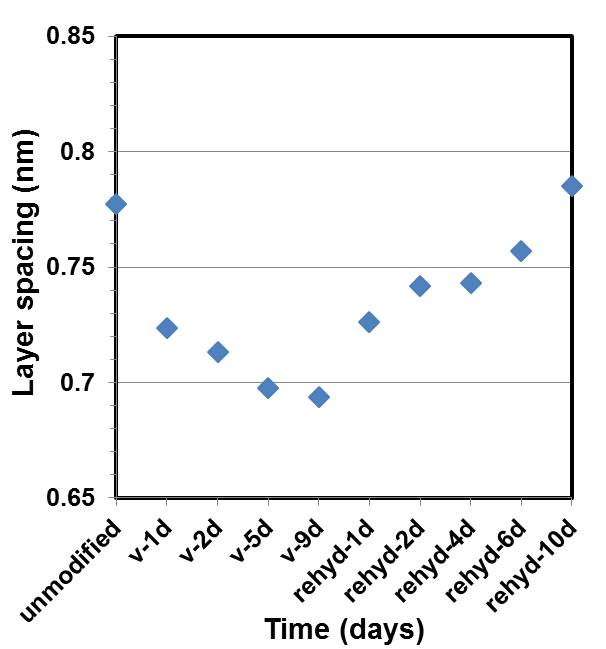 | 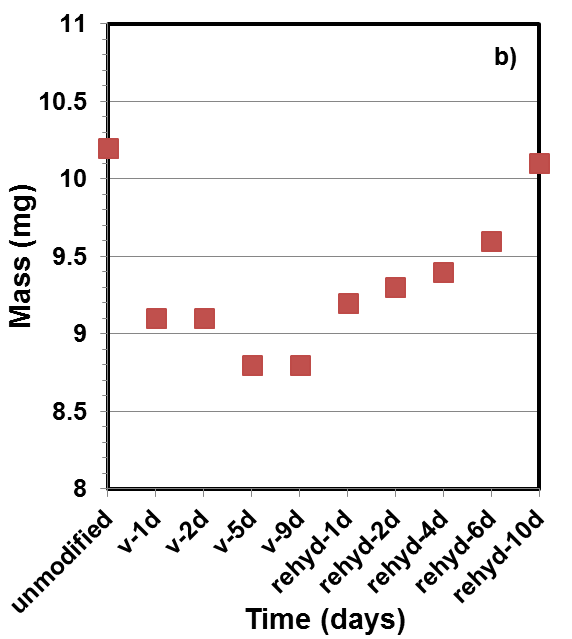 |
| --- | --- |
| 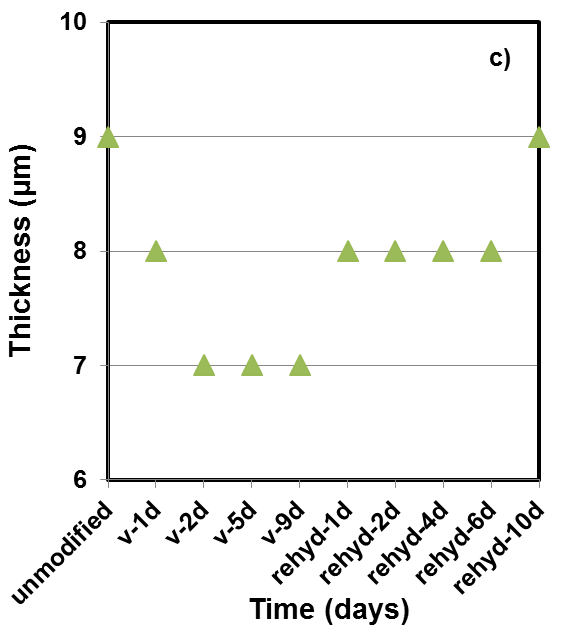 |  |

Figure S1. Experimental results for the changes in a) GO layer spacing in nm, b) mass in mg, and c) thickness in μm of initial (unmodified) sample, samples vacuum dried for 1, 2, 5 and 9 days, and subsequently rehydrated for 1, 2, 4, 6 and 10 days. The change in mass from fully hydrated to vacuum dried for 9 days is about 15%, which is attributed to the water removed.


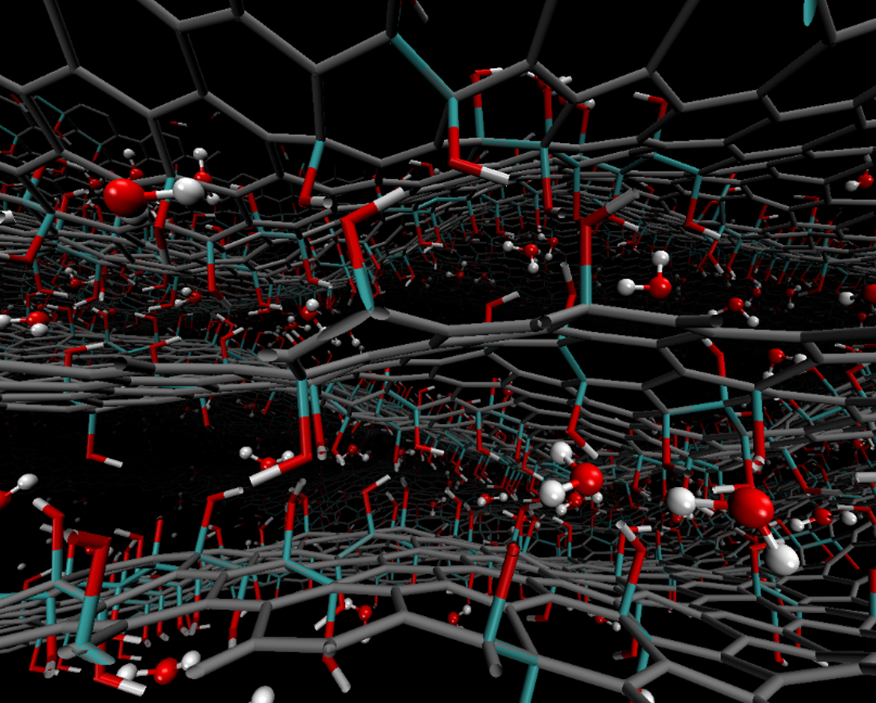


Figure S2. Perspective projection of the GO-water system for 3.9 wt. % water. O, H, C bonded to OH and other C are shown in red, white, teal and grey.

Figure S3. Pair correlation function between hydrogen atom of OH group (H_o_) and oxygen atom of H_2_O (O_w_) for the water content shown in the legend. The first peak occurs at about 0.17 nm and the first minimum is at 0.24 nm.

Figure S4. Pair correlation function between oxygen atom of OH group (O_h_) and hydrogen atom of H_2_O (H_w_) for the water content shown in the legend. The first peak occurs at about 0.17 nm and the first minimum is at 0.24 nm.

Figure S5. The average water cluster size excluding (red square) or including (blue circle) isolated H_2_O molecules for different hydration levels in graphene oxide.
